# Low Power Consumption Nanofilamentary ECM and VCM Cells in a Single Sidewall of High‐Density VRRAM Arrays

**DOI:** 10.1002/advs.201902363

**Published:** 2019-10-07

**Authors:** Min‐Ci Wu, Yi‐Hsin Ting, Jui‐Yuan Chen, Wen‐Wei Wu

**Affiliations:** ^1^ Department of Materials Science and Engineering National Chiao Tung University No. 1001, University Rd., East Dist. Hsinchu City 30010 Taiwan; ^2^ Department of Materials Science and Engineering National United University No. 1, Gongjing Miaoli City Miaoli County 360 Taiwan; ^3^ Center for the Intelligent Semiconductor Nano‐System Technology Research National Chiao Tung University Hsinchu City 30010 Taiwan; ^4^ Frontier Research Center on Fundamental and Applied Sciences of Matters National Tsing Hua University Hsinchu City 30013 Taiwan

**Keywords:** 3D vertical resistive random access memory (VRRAM), high‐density memory arrays, low power consumption, nanofilaments, transmission electron microscope (TEM) structural analysis

## Abstract

The technologies of 3D vertical architecture have made a major breakthrough in establishing high‐density memory structures. Combined with an array structure, a 3D high‐density vertical resistive random access memory (VRRAM) cross‐point array is demonstrated to efficiently increase the device density. Though electrochemical migration (ECM) resistive random access (RRAM) has the advantage of low power consumption, the stability of the operating voltage requires further improvements due to filament expansions and deterioration. In this work, 3D‐VRRAM arrays are designed. Two‐layered RRAM cells, with one inert and one active sidewall electrode stacked at a cross‐point, are constructed, where the thin film sidewall electrode in the VRRAM structure is beneficial for confining the expansions of the conducting filaments. Thus, the top cell (Pt/ZnO/Pt) and the bottom cell (Ag/ZnO/Pt) in the VRRAM structure, which are switched by different mechanisms, can be analyzed at the same time. The oxygen vacancy filaments in the Pt/ZnO/Pt cell and Ag filaments in the Ag/ZnO/Pt cell are verified. The 40 nm thickness sidewall electrode restricts the filament size to nanoscale, which demonstrates the stability of the operating voltages. Additionally, the 0.3 V operating voltage of Ag/ZnO/Pt ECM VRRAM demonstrates the potential of low power consumption of VRRAM arrays in future applications.

## Graphical abstract

A 3D high‐density vertical resistive random access memory (VRRAM) is demonstrated to efficiently increase the device density in this study. The electrochemical migration cell exhibits the potential of the VRRAM application, and the stability of the operating voltages is improved due to the thin Ag sidewall electrode that confines the Ag filaments to a narrow range in the VRRAM structure.

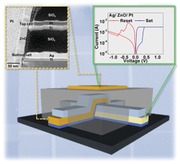

Memory can be divided into volatile memory and nonvolatile memory, where NAND flash is the most widely used nonvolatile storage device due to its rewritable properties and low cost.^1^, ^2^, ^3^ However, some problems exist in NAND flash that need to be solved, such as low writing and reading speed, high operating voltage, and relatively large cell dimensions. To overcome these problems, next‐generation memories, such as resistive random access (RRAM),^4^, ^5^, ^6^ phase change random access memory (PRAM),^7^, ^8^ and magnetoresistive random access memory (MRAM),^9^, ^10^ have been receiving significant attention. Among these next‐generation memories, RRAM is considered as a promising candidate due to its fast switching speed and low power consumption.^11^, ^12^, ^13^, ^14^, ^15^ It also has the potential to achieve high storage densities by building a high‐density crossbar array^16^, ^17^ or constructing a 3D vertical structure.^18^, ^19^, ^20^ A RRAM device is commonly composed of metal–insulator–metal (MIM) structure, which is called “sandwich structure.” There are many types of materials used for the insulator of RRAM, for example, transition metal oxides,^4^, ^6^ solid electrolyte,^5^ and perovskite.^21^, ^22^ The various available options demonstrate the potential applications of a stable structure, low power consumption, and shrinking ability under several nanometers.

While applying voltage to filamentary RRAM device, a conducting path, which is known as conducting filament, appears in the dielectric layer to connect the top and bottom electrodes.^4^, ^5^, ^6^, ^23^ The set process results from the formation of the filaments, while the reset process is caused by the rupture of the conducting filaments. Filamentary resistive switching in RRAM often involves electrochemical migration (ECM) and valance change mechanism (VCM).^6^ In ECM RRAM, the external electric field induces metal ions to migrate from the anode to the cathode and reduces the metal cations into atoms to form a conducting path during the set process. The filaments are dissolved by applying an opposite bias, that is, the negative voltage to the active electrode in the reset process.^4^, ^5^ On the other hand, in VCM RRAM, the oxygen vacancies from the dielectric layer assemble to form filaments in the set process, while in the reset process, the conducting path can be broken by increasing the voltage (unipolar VCM RRAM)^6^, ^24^ or applying a reverse bias (bipolar VCM RRAM).^25^ The operation of ECM RRAM requires a lower power supply and more easily forms/ruptures conducting filaments, but the endurance and stability of the operating voltage are not as excellent as in VCM device.^13^, ^26^ Therefore, we aimed to simultaneously increase the stability of ECM RRAM and build high‐density arrays by introducing a 3D vertical structure.^18^, ^19^, ^20^

Owing to transistors on integrated circuits doubling their quantities every 18 months by Moore's law,^21^ shrinking the device size and maximizing the arrays' density has become significant, with the requirements of an increasing storage density and flash memory gradually developing from 2D NAND to 3D NAND architecture.^3^ To compete with NAND flash and other nonvolatile memories, the development of high‐density RRAM structure is becoming more important. For the purpose of increasing the device density in the vertical direction, scientists have built a 3D vertical RRAM structure (VRRAM).^18^, ^19^, ^20^ However, previous work on the VRRAM structure has focused on VCM RRAM, and there is an absence of studies discussing ECM VRRAM devices. Although ECM RRAM has the advantage of low operating voltage compared to VCM RRAM, it has encountered endurance problems due to the excessively irreversible migration of active atoms in the traditional layered MIM structure.^13^, ^26^ In this work, a specially designed 3D vertical RRAM structure was built, and this structure has the advantages of high‐density, low fabrication cost, and easy build‐up process. The applications of a 3D vertical structure on ECM RRAM could increase the stability of ECM RRAM due to the thin sidewall electrode defining the position of the filaments' formation. Additionally, the evolution of the conducting filaments could be observed in our design, where the conducting filaments in VRRAM play an important role in resistance switching, still requires further investigation. ZnO was selected to be the dielectric,^4^, ^27^ and the sidewall electrodes of VRRAM are Pt and Ag. Each cross‐point of the VRRAM arrays contains one VCM cell and one ECM cell. The fabrication process only required two‐steps of shadow mask deposition to form a two‐layered VRRAM structure, which effectively simplified the fabrication process. The conducting filaments were observed by transmission electron microscope (TEM), and the components of filaments were further analyzed via energy‐dispersive spectroscopy (EDS), fast Fourier transform diffraction patterns (FFT‐DPs), and electron energy loss spectroscopy (EELS). The results showed that the conducting filament is ≈20 nm in diameter, and the shrinkage of interface area between the active electrode and dielectric could effectively confine the dimensions of filament.

The high‐density 3D two‐layered VRRAM cross arrays contained one active electrode and one inert electrode in the sidewall. The schematic in **Figure** **1**a illustrates the structure of the 3D‐VRRAM, where Pt top electrode lines are in the *y*‐direction and Ag bottom electrode lines extend along the *x*‐direction. The VRRAM cross section was obtained in the *XY* plane marked by the white dashed line in Figure 1a (45° between the *x*‐axis and the *y*‐axis, and the cross‐section was viewed in the [110] direction). To measure the top cell (Pt/ZnO/Pt), one of the W probes touched the pillar electrode Pt at the cross‐point and another probe touched the Pt sidewall electrode at the endpoint, as shown in Figure 1b. Therefore, the reactive area was in the ZnO film between the sidewall Pt and pillar electrode. On the other hand, the bottom cell could be operated by applying a voltage between the pillar electrode and Ag sidewall electrode at the endpoint, as shown in Figure 1c. Figure 1d shows the SEM top view image, where the position of two‐layered VRRAM is denoted by the white arrow at the cross‐point. The top electrode line formed an arch‐bridge across the bottom electrode line, as shown in Figure S2 (Supporting Information). The two electrodes would not connect to each other and the sidewall devices could be measured independently at the same cross‐point. The cross‐sectional scanning transmission electron microscopy (STEM) image of the VRRAM is shown in Figure 1e. According to the initial state of the EDS mapping (Figure 1f), two‐layered sidewall electrodes (Pt and Ag electrode) were clearly observed in the 3D‐VRRAM structure, and the dielectric ZnO exhibited a uniform step coverage. The Figure S3 (Supporting Information) shows the cross‐sectional STEM image and EDS mapping of initial state VRRAM structure by introducing the surface cleaning process. The green points and the red points in Figure 1d represent the contact positions of the probe during measurement, which correspond to the operation schematic diagrams in Figure 1b,c, respectively.

**Figure 1:**
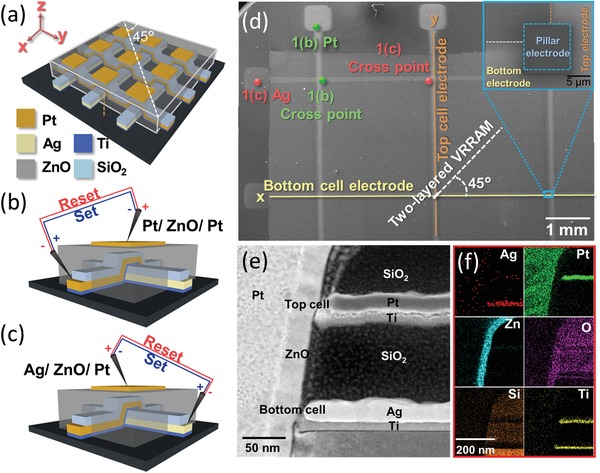
3D two‐layered vertical RRAM arrays. a) The schematic of the 3D‐VRRAM cross array. b,c) The VRRAM operation schematic of Pt/ZnO/Pt (top cell) and Ag/ZnO/Pt (bottom cell). d) The top view SEM image of the 3D‐VRRAM in 3 × 2 array. The top cell and bottom cell of the two‐layered VRRAM appear in 45° at the cross‐point between the sidewall electrode and pillar electrode. The inset shows the enlarged SEM image at cross‐point, and the pillar electrode confines the filament in specific area. The cross‐sectional TEM image of the VRRAM structure cutting from 45° of the cross‐point. f) The EDS mapping of (e).

The forming voltages of VRRAM Ag/ZnO/Pt and Pt/ZnO/Pt are 5.4 and 6.5 V, respectively, as shown in Figure S4 (Supporting Information). Compared to the switching behavior of Pt/ZnO/Pt, the Ag/ZnO/Pt cell requires smaller electric field to conduct the forming process due to the lower electrical potential of Ag and the smaller diffusion coefficient of Ag^+^ in ZnO.^28^ After measuring the top cell, Pt/ZnO/Pt, for several cycles, we found the forming voltage was reduced in the bottom cell, Ag/ZnO/Pt. It is worth mentioning that the operation of the top cell is conductive to the forming process of the bottom cell. The operation of top cell could increase the concentration of oxygen vacancies, leading the Ag ions to more easily diffuse into the ZnO matrix. The cycling *I*–*V* curves of the Ag/ZnO/Pt device are shown in **Figure** **2**a. The set and reset voltages of bottom cell (Ag/ZnO/Pt) are 0.3 and −0.4 V, respectively, which demonstrate the superiority of low operating voltage compared to the Pt/ZnO/Pt cell. As shown in Figure S5 (Supporting Information), When the compliance current decreased, the power consumption could be efficiently reduced. Figure 2b,c show the *I*–*V* curves of Ag/ZnO/Pt in the log(*I*)–log(*V*) (double‐logarithmic plot) scale in set and reset process, respectively. According to fitting lines B2 and B3, the slope of double‐logarithmic plot is ≈1, which means that the current flowing through the dielectric ZnO is proportional to the voltage applied on the cell. The current density of ohmic conduction in the semiconductor can be expressed as

(1) $$J=\sigma E=nq\mu E,\;n=N_{C}\exp\left[-\left(E_{C}-E_{F}\right)/kT\right]$$

where σ is the electrical conductivity, *E* is the electric field acting on the cell, *n* is the number of electrons in the conduction band, *q* is the charge of one electron (1.6 × 10^−19^ C), *µ* is the electron mobility, *N*_C_ is the effective density of states of the conduction band, *k* is the Boltzmann constant, and *T* is the operating temperature. In low resistance state (LRS), the movement of mobile electrons in the conduction band of ZnO causes the occurrence of ohmic conduction.^29^ In high resistance state (HRS) of the Ag/ZnO/Pt cell, ohmic conduction is also observed in low electric field (the slopes of fitting lines B1 and B4a in the double‐logarithmic plot were 1.06 and 1.12, respectively). The ohmic current may appear at a very low voltage in the ZnO films due to a few carriers being generated by thermal excitation, resulting in the conduction current being proportional to the electric field.^30^ In addition, the slope of the double‐logarithmic plot is close to 2 at high electric field in the HRS of Ag/ZnO/Pt, which means the current density *J* is proportional to *V* ^2^. These results correspond to the space‐charge‐limited conduction (SCLC) that followed the ohmic conduction behavior (*I* ∝ *V*) at low electric field and Child's law (*I* ∝ *V* ^2^) at high electric field.^31^, ^32^ The space‐charge‐limited current is caused by the injection of electrons at an ohmic contact of the Ag/ZnO interface. Detailed information on the fitting lines in Figure 2b,c is contained in Figure S6 (Supporting Information).

**Figure 2:**
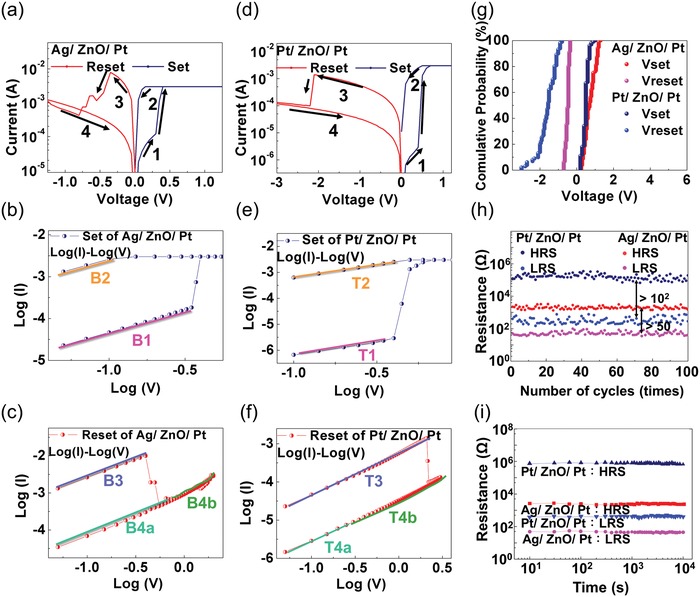
Electrical properties of Ag/ZnO/Pt (bottom cell) and Pt/ZnO/Pt (top cell) in VRRAM. a) Cycling *I*–*V* curves of the Ag/ZnO/Pt device. b,c) The *I*–*V* curves of Ag/ZnO/Pt in the log–log scale in the set and reset process, respectively. d) Cycling *I*–*V* curve in Pt/ZnO/Pt device. e,f) The *I*–*V* curves of Pt/ZnO/Pt in the log–log scale for the set and reset process. g) The threshold voltages distribution of Ag/ZnO/Pt and Pt/ZnO/Pt. h) The endurance test. The results show that both the Ag/ZnO/Pt and Pt/ZnO/Pt cells in VRRAM could operate at more than 100 times. d) The retention measurement. The retention times of both devices are >10^4^ s.

On the other hand, Figure 2d shows the cycling *I*–*V* curves of the Pt/ZnO/Pt device in VRRAM. The set and reset voltages of the top cell (Pt/ZnO/Pt) were 0.5 and −2 V, respectively. The slope of the double‐logarithmic plot (line T2 in Figure 2e and line T3 in Figure 2f) is ≈1 in a low resistance state, which followed the ohmic conduction mechanism, similar to the Ag/ZnO/Pt device. After Pt/ZnO/Pt switched back to the HRS, the slope of the fitting line T4a in double‐logarithmic plot is 1.02 at a low *V* region, demonstrating that it is also conducted by ohmic conduction.^29^, ^30^ Then, the fitting line T4b revealed a linear characteristic of ln (*I*/*V*) versus *V* ^1/2^ plot in the high voltage region of HRS, which corresponds to Poole–Frenkel emission (P–F emission),^33^ as shown in Figure S4f (Supporting Information). During the forming process, a small amount of electrons in poly‐crystallized ZnO could be excited to the conduction band by thermal energy, and these excited electrons are movable in the crystal. While in the high voltage region, a large amount of electrons could get into the conduction band of ZnO due to the contribution of a large electric field for the activation energy. The high voltage region provides a large electric field, which leads to the P–F emissions dominating the conduction mechanism. Detailed information on the fitting lines in Figure 2e,f is contained in Figure S7 (Supporting Information).

We further measured the endurance, retention time and threshold voltages distributions of the Pt/ZnO/Pt and Ag/ZnO/Pt cells and found that both cells displayed good electrical properties, especially the bottom cell. The threshold voltages distribution of Ag/ZnO/Pt and Pt/ZnO/Pt (Figure 2g) presented the stability of switching voltages. The set voltages of ECM Ag/ZnO/Pt ranged in 0.7 V (from 0.3 to 1 V), and the reset voltages were distributed in 0.4 V (from −0.4 to −0.8 V). As comparison, a traditional layered Ag/ZnO/Pt RRAM was fabricated and measured. The set and reset voltages ranged from 0.3 to 1.75 V and from −0.35 to −1.2 V, respectively, as shown in Figure S8 (Supporting Information). This demonstrated that the importing of the VRRAM architectures can lead the operating voltages of the ECM cell to be more concentrated, even reaching the stable level of the VCM cells. Moreover, as shown in Figure 2h, the endurance test indicated that both cells could operate for more than 10^2^ cycles. By the application of vertical structure, Ag/ZnO/Pt VRRAM has stable resistance switching, where the cell could continue operating without degradation, as shown in Figure S9 (Supporting Information). The optimized endurance and stable operating voltage of the bottom cells were attributed to the thin film electrode in the VRRAM structures, and these results were verified later in the article. Thus, the retention test in Figure 2i indicates that both Pt/ZnO/Pt and Ag/ZnO/Pt could maintain their resistance states for a long time after switching, and the retention time could exceed 10^4^ s.

To realize the reaction of resistance switching, the top cell sidewall cross section prepared by the focused ion beam, was observed via transmission electron microscope after measured it for 30 cycles. **Figure** **3**a shows the STEM image of the top cell sidewall interface after cycling. In the STEM image, the ZnO layer exhibited a dark‐contrast sandwiched between the sidewall electrode and the pillar electrode, and there was a white path among the ZnO layer marked by white dashed lines. We considered this white path as the conducting filament that led the device to switch into LRS. The obviously white path between the two electrodes in another top cell was also observed in the STEM image in Figure S10b (Supporting Information). According to the energy‐dispersive spectrometer (EDS) point analysis in Figure 3b, the Zn/O ratio was ≈0.39 in the region away from the conducting path (point 2). However, the ratio of Zn/O increased to 1.35 at the path (point 1), which demonstrates that the path contained less oxygen than the other ZnO region. The electron energy loss spectroscopy (EELS) line scan (Figure 3c) also provided information about the oxygen vacancies conducting filament. The EELS line scan corresponded to the white line, which indicated that the composition of oxygen in the conducting filament region was less than that in the ZnO matrix (between the SiO_2_ barrier and pillar electrode). Moreover, the EELS spectra of the Zn L edge in Figure 3d, composed of the Zn L peak at 1020 eV, demonstrated that the peak shape was slightly different between the ZnO matrix and ZnO_1−_ *_x_* filament. The appearance of a sharp peak in regions a and d in the spectra of the ZnO matrix represented that the Zn^2+^ bonded with O^2−^. However, in the spectrum of the filament, there was no raised peak in region b. The smooth curve in region b implied a reduction of oxygen, which resulted from the bonding in the filament region being similar to pure Zn metal.^34^, ^35^, ^36^, ^37^ This evidence pointed out that the conducting path in Pt/ZnO/Pt VRRAM was composed of oxygen vacancies. The generation and migration of oxygen vacancies in ZnO would be thermally driven, induced by electric field. As shown in Figure S11a (Supporting Information), the forming voltage increased with the decreasing sweeping rate owing to the less energy supply at the same time interval. In addition, based on Figure S11b (Supporting Information), LRS resistance decreased with the increasing temperature, demonstrating the filaments were composed of oxygen vacancies. Figure 3e shows the schematic of the switching mechanism, where the oxygen vacancies would generate a conducting path in the ZnO layer due to the influence of the electric field. The oxygen vacancies acted as the traps and grabbed electrons; therefore, the electrons could cross the ZnO layer along the oxygen vacancies path. In the reset process, the filaments could be broken by Joule heating effect due to a large current; therefore, Pt/ZnO/Pt has larger reset voltage, as shown in Figure 2d.

**Figure 3:**
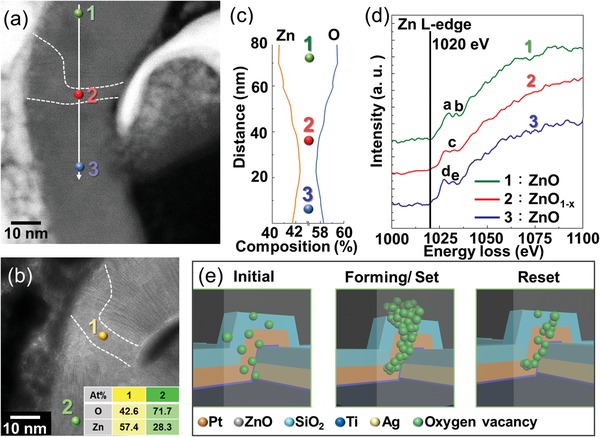
The component analysis of the top cell (Pt/ZnO/Pt) and the schematic of the mechanism. a) STEM dark‐field image of the operated VRRAM top cell (Pt/ZnO/Pt). At point 2, a white path connects the sidewall electrode to the pillar electrode. b) TEM bright‐field image of the top cell. The inset table in (b) shows the EDS point analysis at points 1 (in filaments) and 2 (ZnO matrix). c) The elementary composition derived from the EELS line scan within the white solid line in (a). d) The EELS spectra of the Zn L‐edges collected from the ZnO matrix (regions 1 and 3) and ZnO_1−_ *_x_* filaments (region 2). e) Schematic of the switching mechanism in the top cell (Pt/ZnO/Pt). The oxygen vacancies play an important role in the resistance switching of VCM RRAM.

Next, we measured the bottom cell for several cycles and maintained it in the LRS for the purpose of clarifying which factors dominated the switching phenomenon in the bottom cell and why the 3D vertical ECM RRAM possessed enhanced endurance, stable threshold voltage distribution, and low forming voltage. Figure S12 (Supporting Information) shows the STEM image of the entire sidewall after cycling test. The ZnO layer near the top sidewall electrode maintained its morphology, while the morphology changed near the bottom sidewall electrode due to the migration of the active Ag electrode. **Figure** **4**a shows the TEM image of the bottom cell sidewall interface after the cycling test. Unlike the STEM image, the ZnO layer exhibited a light‐contrast sandwiched between the dark contrast of the electrodes, which are marked by white dashed lines. In Figure 4a, we can clearly observe that a 20 nm width dark path connected the sidewall Ag electrode to the pillar Pt electrode in the bottom cell. The dark path also appeared in another cycled VRRAM bottom cell, as shown in Figure S13 (Supporting Information). The dark path was regarded as the conducting filament, which resulted in the low resistance state of the bottom cell. Therefore, we analyzed the composition of the filament by use of EDS. According to the EDS mapping in Figure 4b, the Ag distribution exactly matched the conducting path, demonstrating that the filament consisted of Ag. The inset in Figure 4c is the FFT pattern converted from the Ag filament HRTEM image in Figure S14 (Supporting Information). The TEM dark‐field images in Figure 4d,e were acquired from the diffraction spots of the Ag (200) plane and the Ag (111) plane, as highlighted by the red lined circles d and e, respectively. The EDS mapping, FFT patterns and SADE results demonstrated that the conducting filament is composed of polycrystalline Ag. As shown in the schematic of Figure 4f, in the forming or set process, the Ag sidewall electrode was the anode and the Pt pillar electrode served as the cathode. The Ag atoms in the sidewall electrode were ionized into Ag^+^ ions and diffused through ZnO to the cathode in the electric field. After the Ag^+^ ions arrived at the cathode, they accepted electrons and were reduced into Ag atoms. These reduced Ag atoms accumulated near the cathode and eventually connected to the sidewall electrode and the pillar electrode. The formation of the Ag filaments caused the device to switch into LRS during the forming or set process. Because of the applied voltage within the electrodes, the electric field provided thermal energy to generate small amounts of oxygen vacancies, and these vacancies were beneficial to Ag migration. While in the reset process, a negative bias was applied to the Ag electrode and the pillar electrode was grounded. The Ag atoms near the anode in the filament were ionized and migrated away from the pillar electrode. The diffusions of the Ag^+^ ions caused a rupture of filaments, which led the bottom cell to switch back to the HRS. Therefore, the active metal Ag plays an important role in the resistance switching in the electrochemical migration (ECM) switching mechanism.

**Figure 4:**
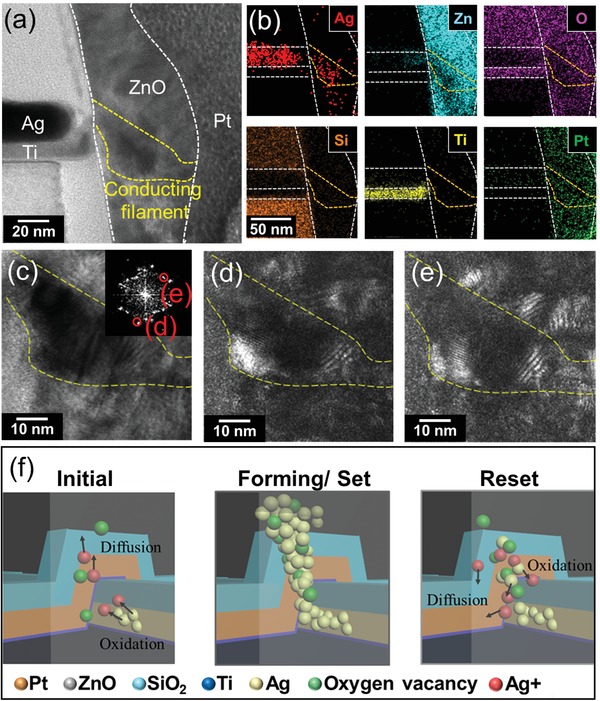
The component analysis of the bottom cell (Ag/ZnO/Pt) and the schematic of the mechanism. a) TEM image of the Ag/ZnO/Pt VRRAM cell (bottom cell) after operating for 30 cycles. b) EDS mapping of various elements corresponding to (a). The silver elements distribution in the ZnO layer demonstrates the diffusion of silver. c) Bright field TEM image of the conducting filament in ZnO. d,e) Dark‐field TEM image acquired from the selected area electron diffraction (SAED) of d) the Ag {200} planes and e) the Ag {111} planes, corresponding to the inset FFT pattern of (c). f) Switching mechanism of the Ag/ZnO/Pt device. Upon applying a voltage to the Ag sidewall electrode, and when the Pt electrode pillar is grounded, silver atoms in the sidewall electrode would oxidize into silver ions and diffuse into ZnO. The diffusing silver ions would reduce near the cathode during the forming or set process to form a conducting path.

The reduced endurance and unstable threshold voltages in the ECM cell suffer from the expansion of active atoms filaments. In the VRRAM structure, the 40 nm thin film sidewall electrode effectively reduced the contact area of the Ag electrode and the ZnO dielectric, so the Ag filaments could be confined in a narrow range around the Ag electrode and then the dimensions of filaments were reduced (the conducting filament is only 20 nm in Figure 4a). Consequently, the stability of the operating voltages could be enhanced. The deterioration of the HRS also decreased due to the narrow filaments, so the endurance of the ECM bottom cell could be enhanced. The conducting filaments in the bottom cell are mainly composed of Ag atoms with few oxygen vacancies. According to Figure 3, the filaments of the VRRAM top cell consisted of oxygen vacancies that were generated during the influence of the electric field. After repeatedly cycling the top cell, the ZnO layer became rich in localized oxygen vacancies, so the Ag atoms did not require too much energy to diffuse through the oxygen‐deficient regions. The forming voltage then decreased.

In summary, we successfully fabricated 3D high‐density VRRAM arrays. In this novel structure, the sidewall device contained different VRRAM types, including VCM and ECM cells. According to the *I*–*V* characteristics, we can conclude that the conduction mechanisms in the LRS of both Pt/ZnO/Pt and Ag/ZnO/Pt VRRAM were ohmic conduction. In HRS, the Ag/ZnO/Pt cell was conducted by SCLC, while the Pt/ZnO/Pt was dominated by ohmic conduction at a low electric field and P–F emission at a high electric field. In addition to the fitting results, we also provided evidence of the filaments' composition, information on the adjacent interference, and the switching mechanism in both cells. The top Pt/ZnO/Pt cell had oxygen vacancy filaments between the electrodes and behaved with a VCM switching mechanism, while the filaments in the bottom Ag/ZnO/Pt cell were composed of Ag atoms. The repeated operation of the top VCM cell would reduce the forming voltage of the bottom ECM device because the Ag^+^ ions would more likely migrate into the oxygen‐deficient regions of the ZnO matrix. The thermal effect in the VCM cell may affect the adjacent cells, which should be dealt with through the device design. The ECM cell exhibited the potential of the VRRAM application, and the stability of the operating voltages could be improved due to the thin Ag sidewall electrode in the VRRAM structure. The shrinkage of the sidewall electrode could reduce the contact area of the Ag and ZnO layer effectively, which confines the Ag filaments to a narrow range. This novel structure can be applied to 3D integration and neuromorphic computing systems.

## Experimental Section

To establish our VRRAM cross arrays, Ti (10 nm)/Ag (40 nm)/SiO_2_ (100 nm) was first deposited on Si substrate through a shadow mask via e‐gun evaporation to form the column lines, and the SiO_2_ between them served as an insulating barrier between the two sidewall electrodes. Next, the mask was rotated by 90° in order to cross over the predeposited bottom cell, and Ti (10 nm)/Pt (40 nm)/SiO_2_ (250 nm) was deposited to form the row lines. Then, 50 nm of ZnO was deposited by RF‐sputter to cover all of the crossing points, with each cross‐point containing a bottom cell with Ag sidewall electrode and a top cell with Pt sidewall electrode. The bare Pt and Ag electrodes at the endpoint of each line were uncovered for probe contact. Finally, 100 nm thick Pt was deposited on the top of ZnO to form the pillar electrode. Detailed information about the preparation method is contained in Figure S1 (Supporting Information).

To realize the switching mechanism of two different types of devices, focus‐ion beam (FIB) techniques were used to fabricate the TEM sample by slicing the cross‐point of the VRRAM arrays at 45°. A transmission electron microscope was used to observe the initial state and low resistance state in both cells, and EDS was used to analyze the filamentary compositions. Additionally, the oxygen vacancies in ZnO were identified by the EELS spectra.

## Conflict of Interest

The authors declare no conflict of interest.

## Supporting information

Supplementary
