# Supplementary material for: Low Power Consumption Nanofilamentary ECM and VCM Cells in a Single Sidewall of High‐Density VRRAM Arrays
Source: Adv Sci (Weinh). 2019 Oct 7;6(24):1902363. doi: 10.1002/advs.201902363 (PMC6918122; doi:10.1002/advs.201902363)
Supplement: Supplementary file 1 — Supplementary [file ADVS-6-1902363-s001.pdf]

Copyright WILEY-VCH Verlag GmbH & Co. KGaA, 69469 Weinheim, Germany, 2019.

ADVANCED SCIENCE
Open Access

Supporting Information

for *Adv. Sci.,* DOI: 10.1002/advs.201902363

Low Power Consumption Nanofilamentary ECM and VCM Cells in a Single Sidewall of High-Density VRRAM Arrays

Min-Ci Wu, Yi-Hsin Ting, Jui-Yuan Chen, and Wen-Wei Wu**

Copyright WILEY-VCH Verlag GmbH & Co. KGaA, 69469 Weinheim, Germany, 2019.

Supporting Information

Low Power Consumption Nanofilamentary ECM and VCM Cells in a Single Sidewall of High Density VRRAM Arrays

*Min-Ci Wu, Yi-Hsin Ting, Jui-Yuan Chen, and Wen-Wei Wu**

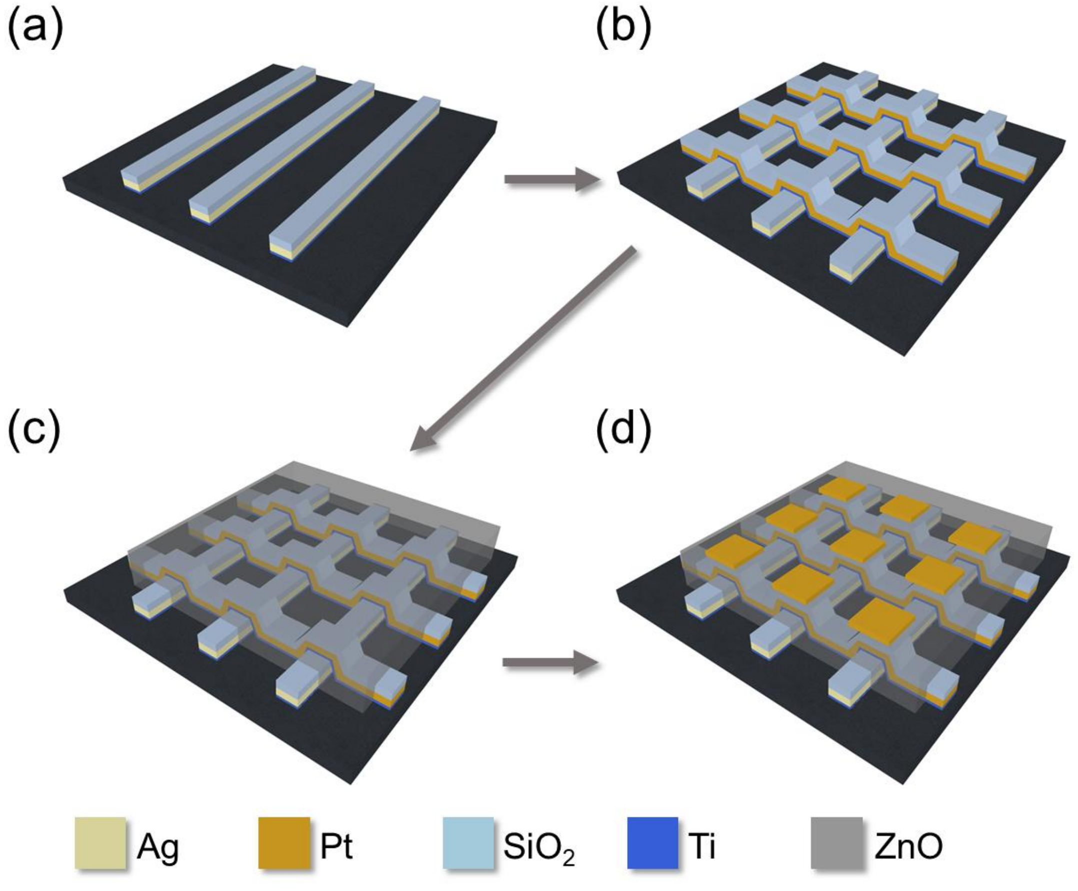

Figure S1. Fabrication process of three-dimensional vertical-RRAM structures. (a) $Ti/Ag/SiO_2$ electrodes lines with 15 nm/40 nm/100 nm thicknesses and 100 μm width were deposited via e-gun evaporation through a metal mask. (b) $Ti/Pt/SiO_2$ electrodes lines with thicknesses of 15 nm/40 nm/100 nm and 100 μm width were deposited via e-gun evaporation

through a rotated 90-degree metal mask. The Ti/Pt/SiO_2 lines cross over the Ti/Ag/SiO_2 lines to form the electrodes array in the VRRAM structure. (c) 50 nm ZnO were deposited via RF-sputter to cover the electrodes array. (d) 100 nm Pt were deposited and defined at cross point via e-gun evaporation.

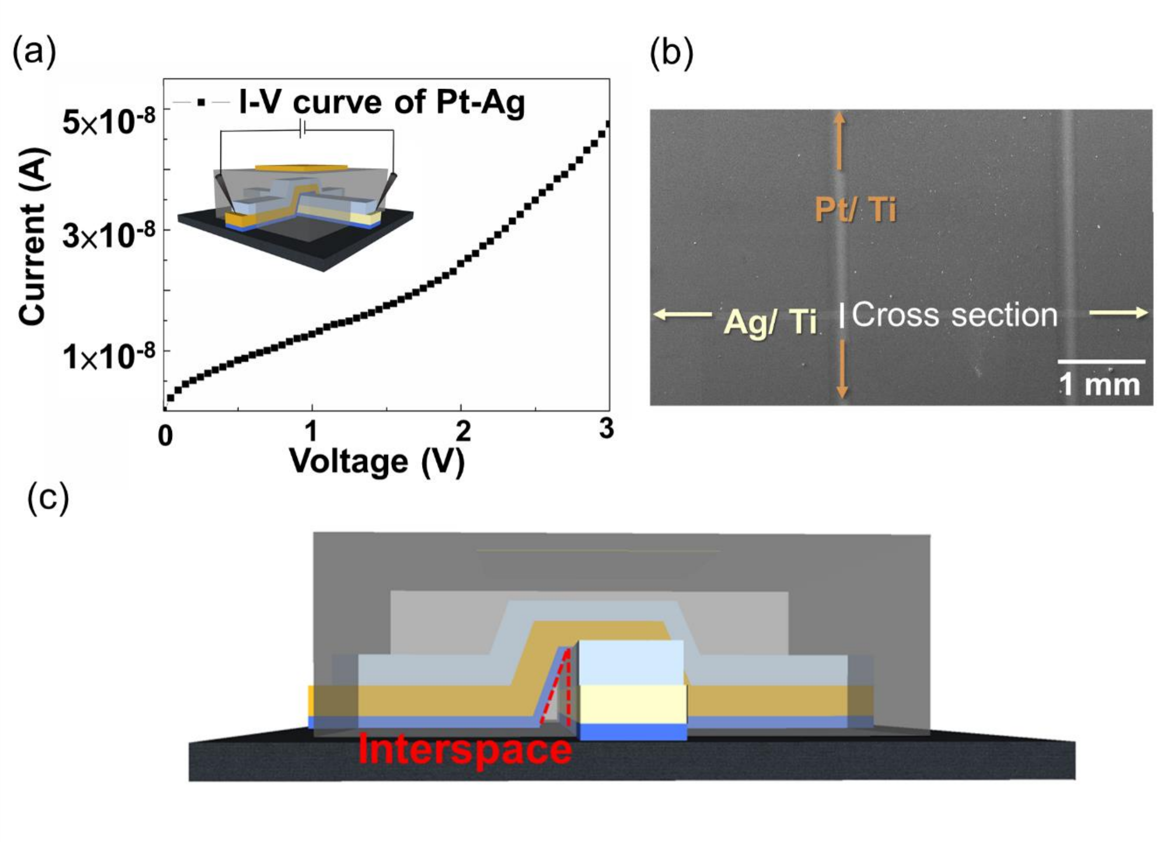

Figure S2. The contact test of Ag/Ti and Pt/Ti electrodes. (a) The I-V characteristic of Ag/Ti and Pt/Ti electrodes lines at a cross point, demonstrating that the Pt/ Ti and Ag/Ti do not contact to each other at cross point. (b) The top view SEM image of VRRAM. The location of cross-sectional schematic in (c) was denoted in white line. (c) The schematic of the Pt/Ti crossover the Ag/Ti at cross point. An interspace was formed between two electrodes, the Pt/ Ti and Ag/Ti did not directly contact to each other at cross point.

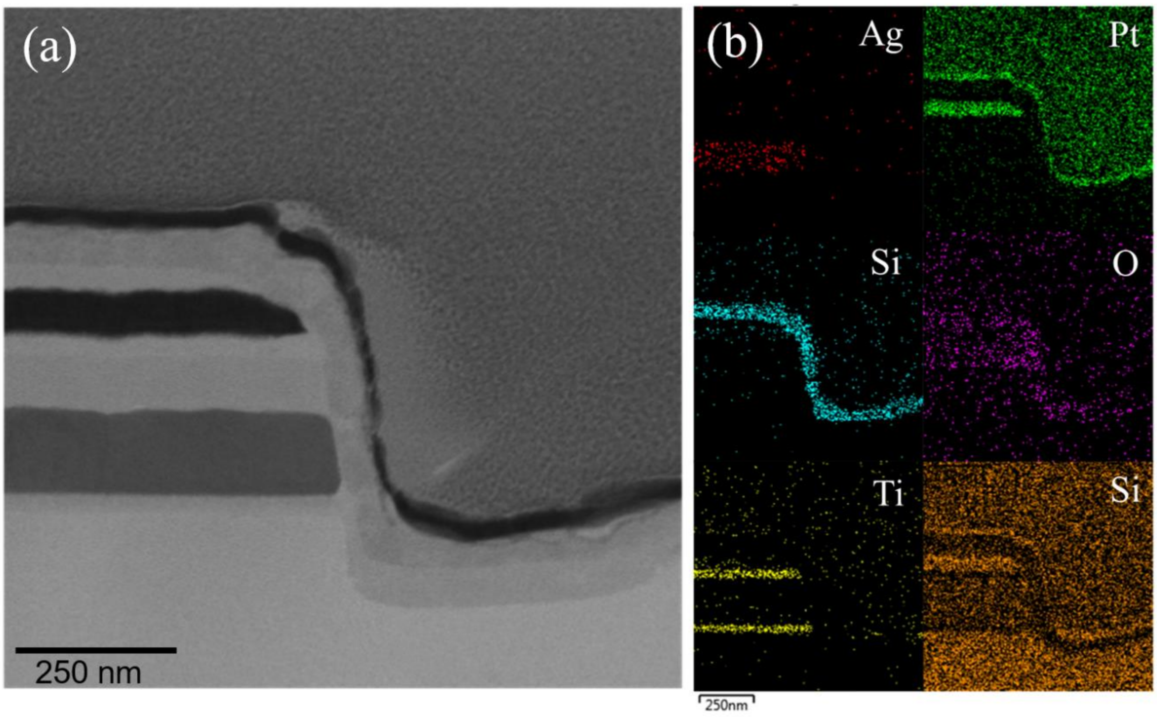

Figure S3. (a) The STEM dark-field image of the sidewall structure after surface cleaning. (b) EDS mapping of (a). The forming voltage of this device was similar to the device in Figure 1. The non-clear surface in Figure 1(e) might result from the FIB process.

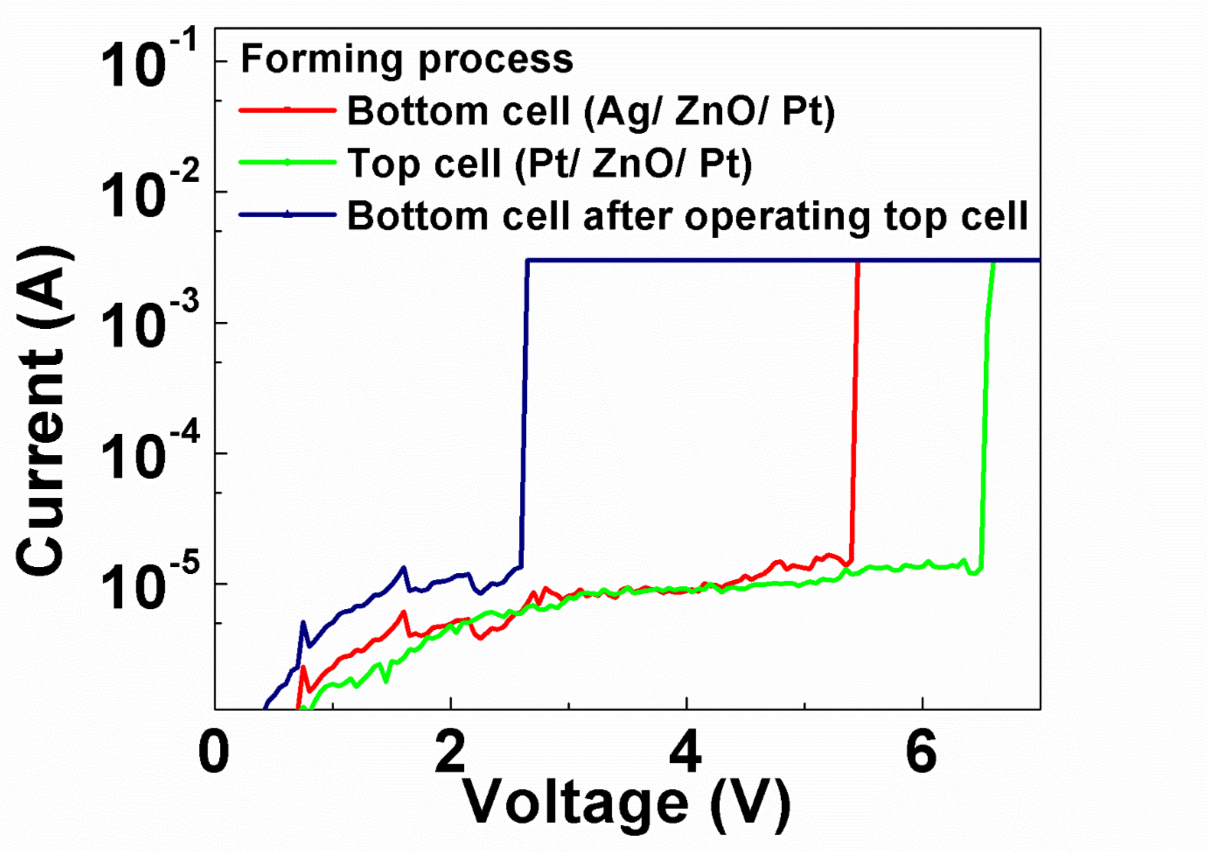

Figure S4. The forming processes of both the VCM and ECM cells. The red and green curves are the original forming curves, and the blue curve is the forming curve of the bottom cell after repeatedly operating the top cell. The thermal effect in the VCM cell would affect the adjacent ECM cells.

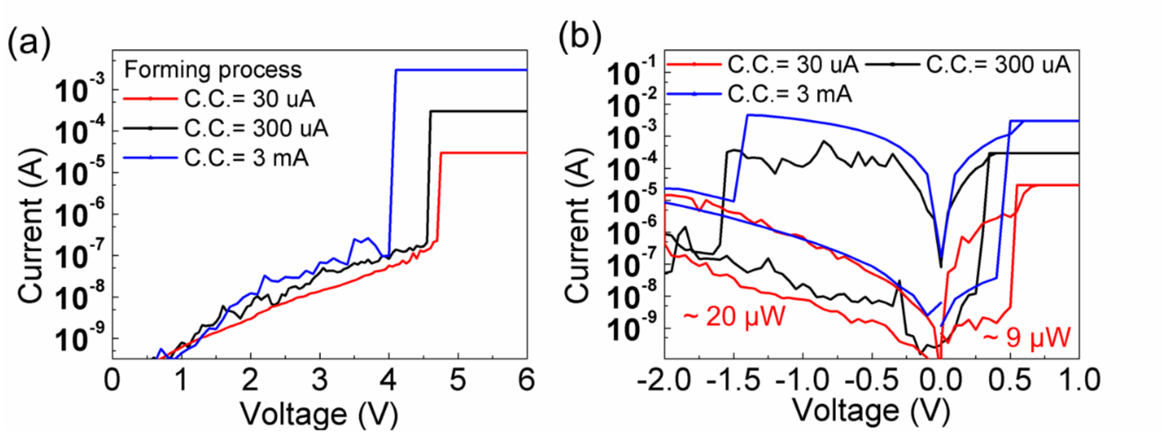

Figure S5 The I- V characteristics of Ag/ZnO/Pt device operation in different compliance current. (a) The forming process of Ag/ZnO/Pt cells with different compliance current: 30 μA, 300 μA and 3 mA. (b) The I-V curves of Ag/ZnO/Pt in different compliance current: 30 μA, 300 μA and 3 mA. When the compliance current decreased, the power consumption could be efficiently reduced. Decreasing the compliance current to 30 μA, the power consumption could be reduced to 9 μW and 20 μW in set and reset process, respectively.

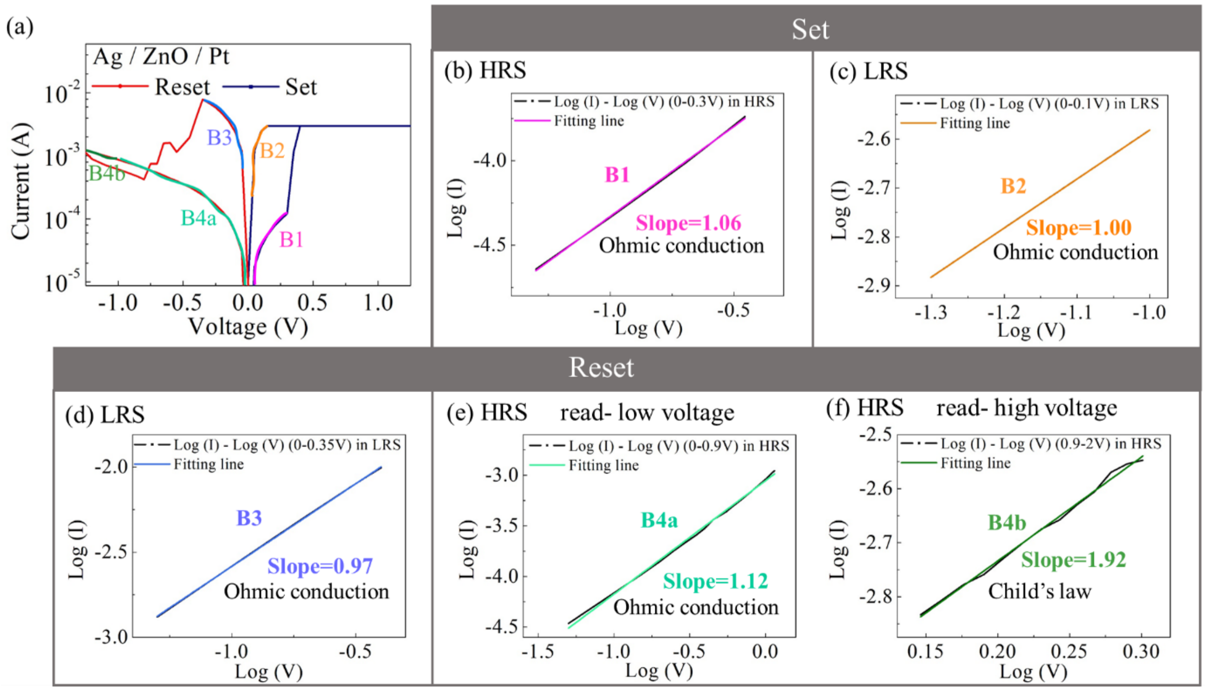

Figure S6. The IV characteristics and fitting results of the bottom cell (Ag/ZnO/Pt). (a) The I-V curves of the Ag/ZnO/Pt cell. (b-c) Double-logarithmic plot in the HRS and LRS of the set process. (d-f) Double-logarithmic plot in the LRS and HRS of the reset process. In the LRS, the slopes in a double-logarithmic scale are 1.00 and 0.97, so the mechanism is ohmic conduction. In the HRS, the slopes in a double-logarithmic scale are 1.06 and 1.12 in the low voltage region and 1.92 in the high voltage region. Therefore, the conduction in the HRS is dominated by SCLC.

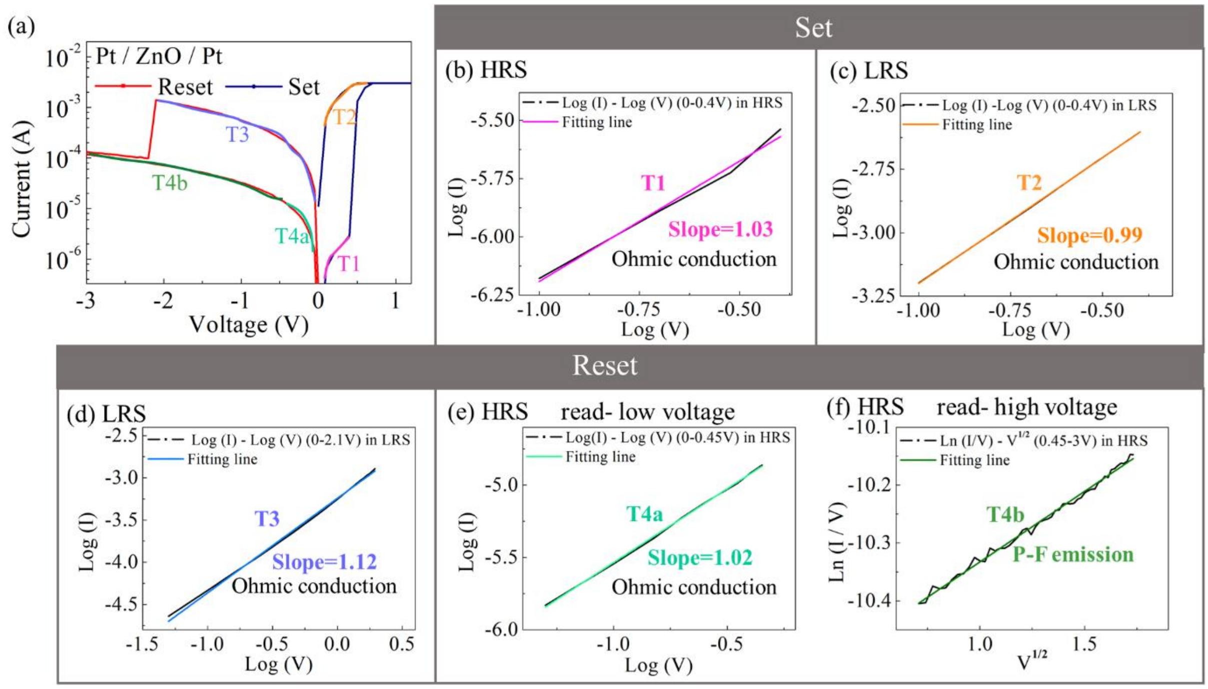

Figure S7. The IV characteristic and fitting results of the top cell (Pt/ZnO/Pt). (a) The I-V curves of the Pt/ZnO/Pt cell. (b-c) Double-logarithmic plot in the HRS and LRS of the set process. (d) Double-logarithmic plot in the LRS of the reset process. (e-f) Double-logarithmic plot under a low voltage and ln (I/V) versus $V^{1/2}$ plot under a high voltage. In the LRS, the slopes in a double-logarithmic plot are 0.99 and 1.12, so the mechanism is ohmic conduction. In the HRS, the slopes in a double-logarithmic plot are 1.03 and 1.02 in the low voltage region, and the relationship of ln (I/V) and $V^{1/2}$ is linear in the high voltage region. Therefore, the conduction in the HRS is dominated by ohmic conduction at a low voltage and P-F emission at a high voltage.

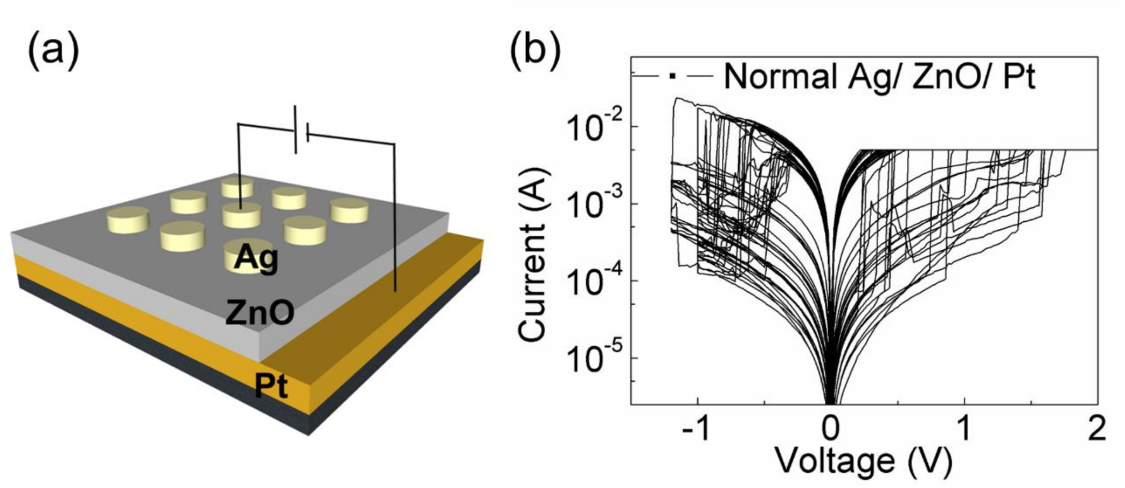

Figure S8. The normal MIM Ag/ZnO/Pt RRAM as the comparison with Ag/ZnO/Pt cell in 3D vertical structure. (a) Schematic of Ag/ZnO/Pt normal device. (b) The I-V curves of normal Ag/ZnO/Pt cell for 40 cycles. The set voltage of normal Ag/ZnO/Pt range from 0.3 to 1.75 V, and the reset voltage range from -0.35 to -1.2 V. This results demonstrated that the threshold voltage of our vertical Ag/ZnO/Pt is more stable than normal device.

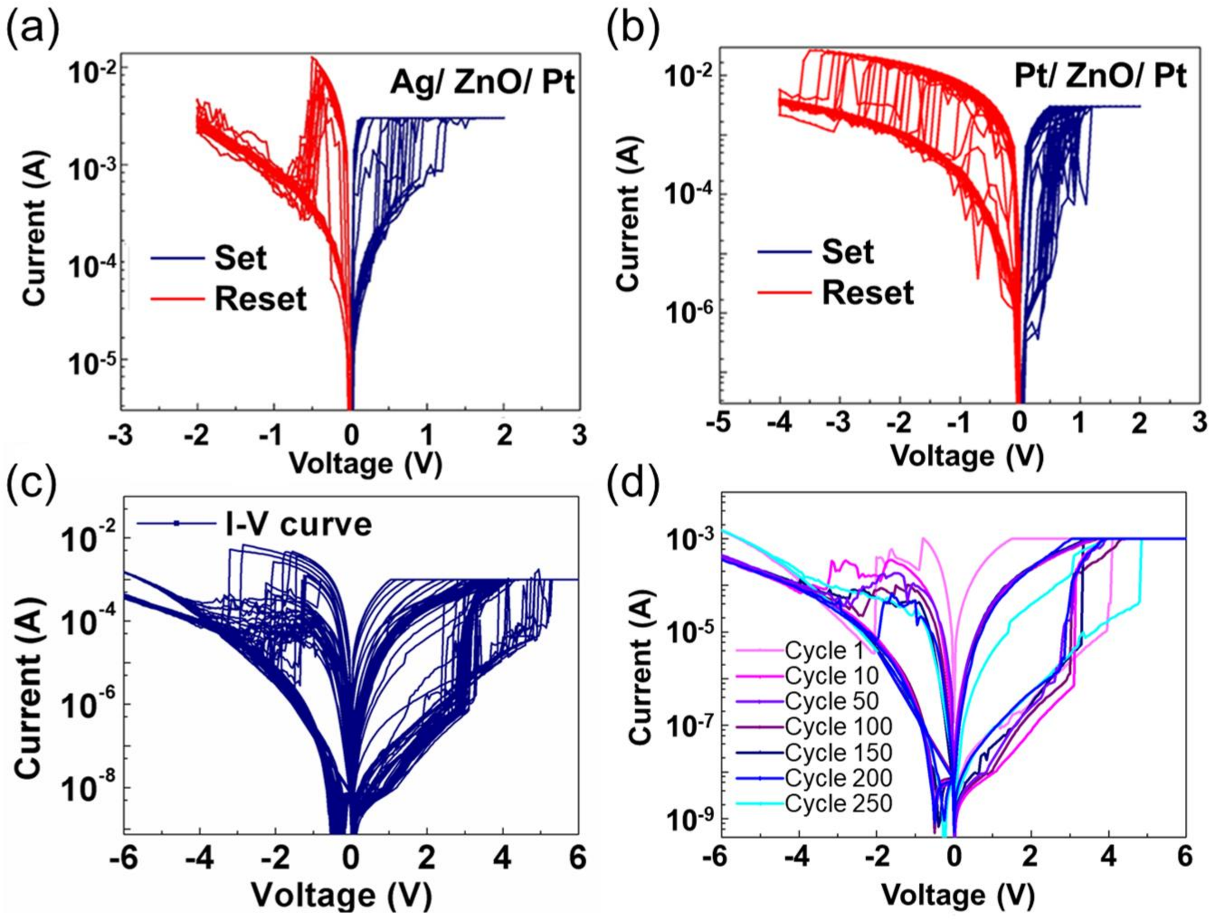

Figure S9. Cycling measurements of the Pt/ZnO/Pt cell and the high-endurance Ag/ZnO/Pt cell. (a) 100 cycles of the bottom cell (Ag/ZnO/Pt). (b) 100 cycles of the top cell (c) another bottom cell that could operate more than 250 times. (d) Cycle 1, 10, 50, 100, 150, 200, and 240 of (c).

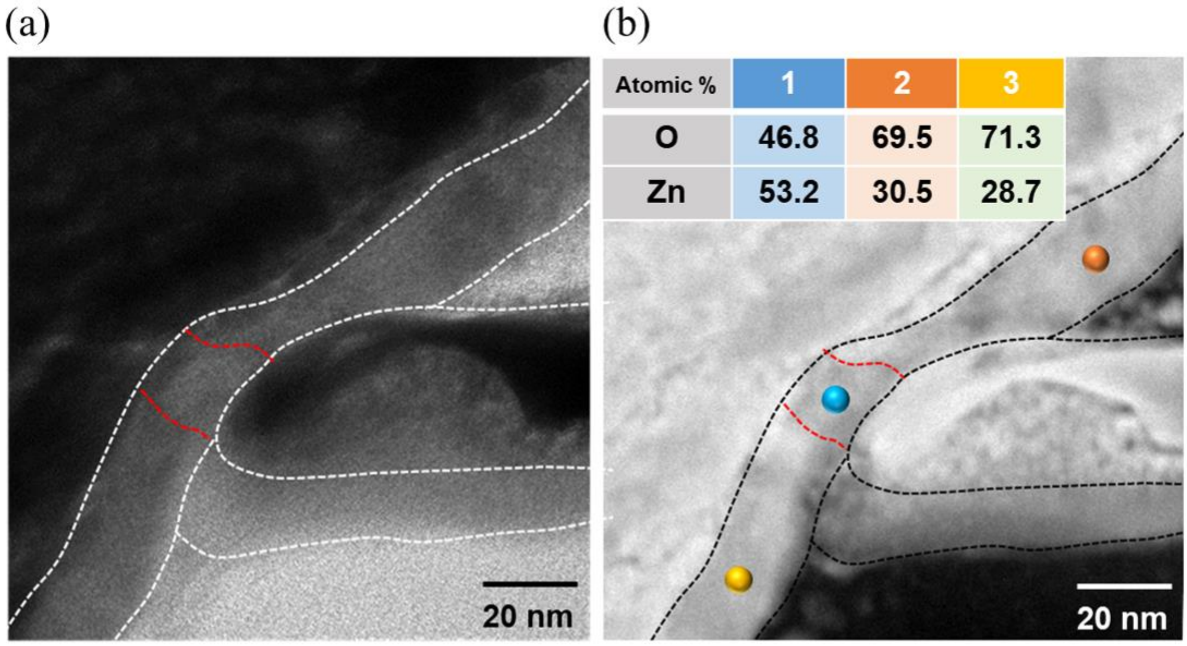

Figure S10. The TEM, STEM images and EDS point analysis of the oxygen filaments in another Pt/ZnO/Pt cell. (a) TEM image of VRRAM. The Pt/ZnO/Pt cell (top cell) was operated for 30 cycles. (b) The STEM image and EDS point analysis. The conducting path at point 1 contains a lower atomic percent of oxygen than the other points, which are far away from the sidewall electrode.

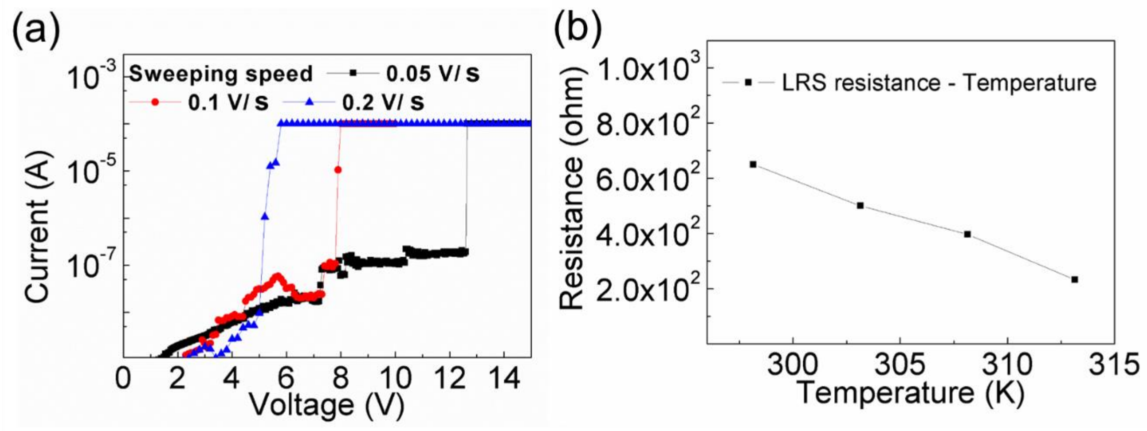

Figure S11. (a) I-V curve of Pt/ZnO/Pt forming process with different sweeping speed 0.05 V/s, 0.1 V/s and 0.2 V/s. While the sweeping speed decreased, the Pt/ZnO/Pt cell obtains less energy in the same time interval, so it requires larger forming voltage to generate a sufficient amount of oxygen vacancies. (b) The plot of Pt/ZnO/Pt LRS resistance varied with temperature. The LRS resistance decreased as the increasing temperature, which demonstrated the filaments were composed of oxygen vacancies.

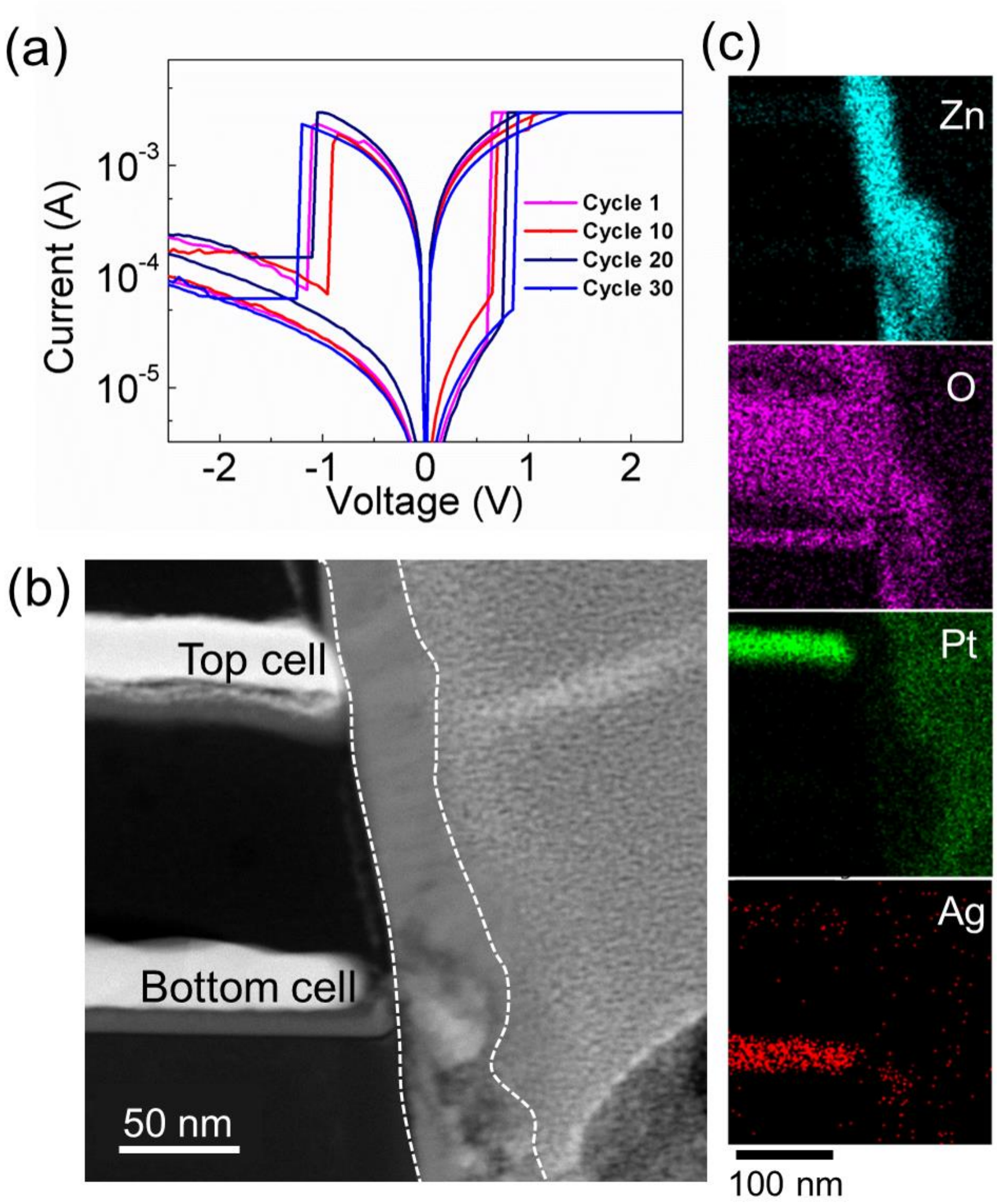

Figure S12. The morphology of entire sidewall structure after several cycles measurement of bottom cell Ag/ZnO/Pt. (a) The I-V curve of 30 cycles Ag/ZnO/Pt cell operation. (b) The STEM image of the entire sidewall after measuring 30 cycles. (c) The EDS mapping. The ZnO around bottom sidewall electrode changed after operating bottom cell for 30 cycles, but the sidewall around top cell without operating maintained its morphology well.

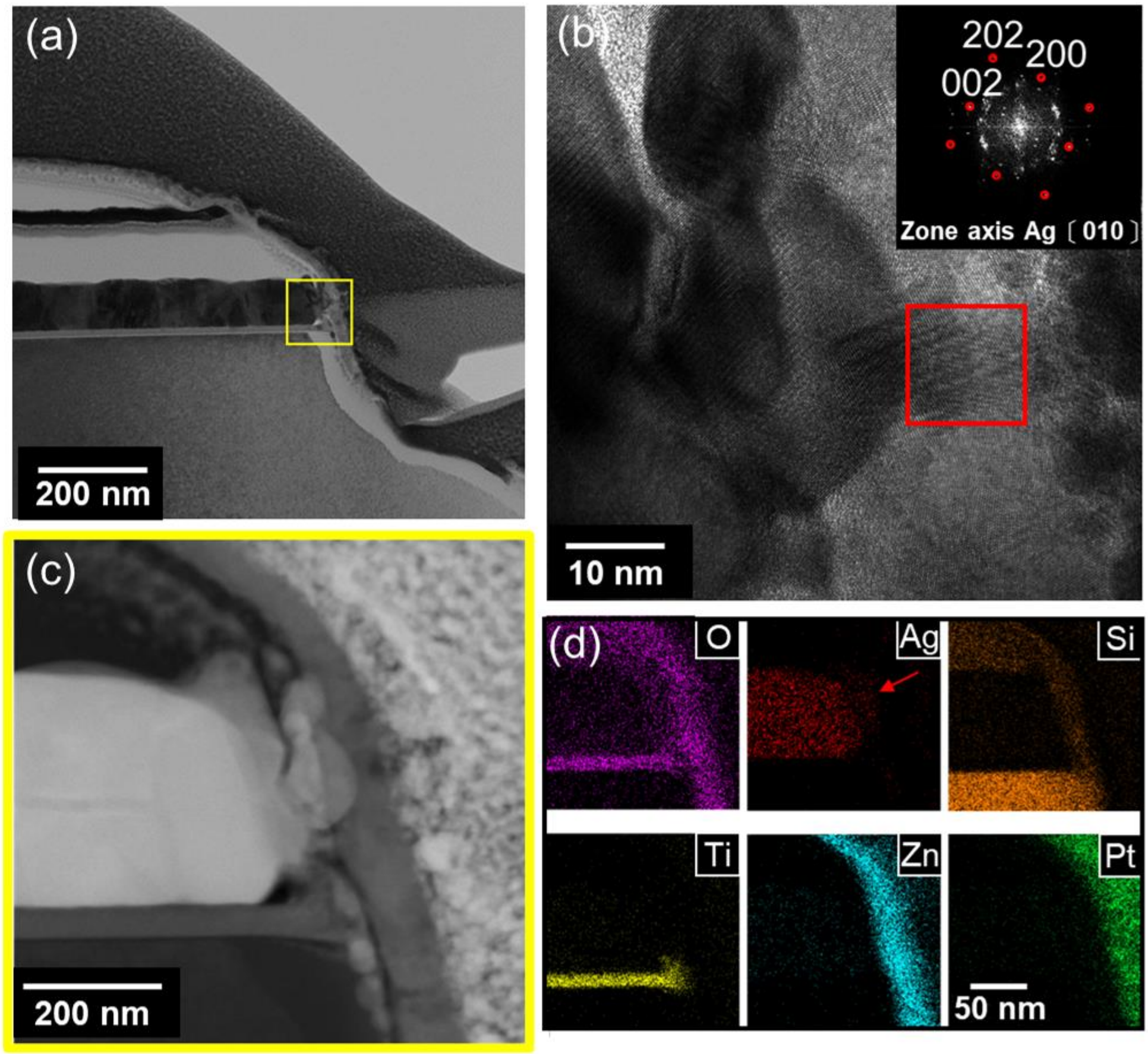

Figure S13. The TEM, STEM images and EDS mapping of Ag filaments in another Ag/ZnO/Pt cell. (a) The TEM image of VRRAM. The Ag/ZnO/Pt cell (bottom cell) was operated for 30 cycles. (b) The high-resolution TEM image of the bottom cell electrode Ag and the sidewall dielectric ZnO interface. The inset is the FFT pattern of the red frame, and the FFT pattern shows that Ag diffused into ZnO. (c) The STEM dark-field image of the sidewall structure. (d) EDS mapping of (c). The silver elements distribution in the ZnO layer demonstrates the diffusion of silver.

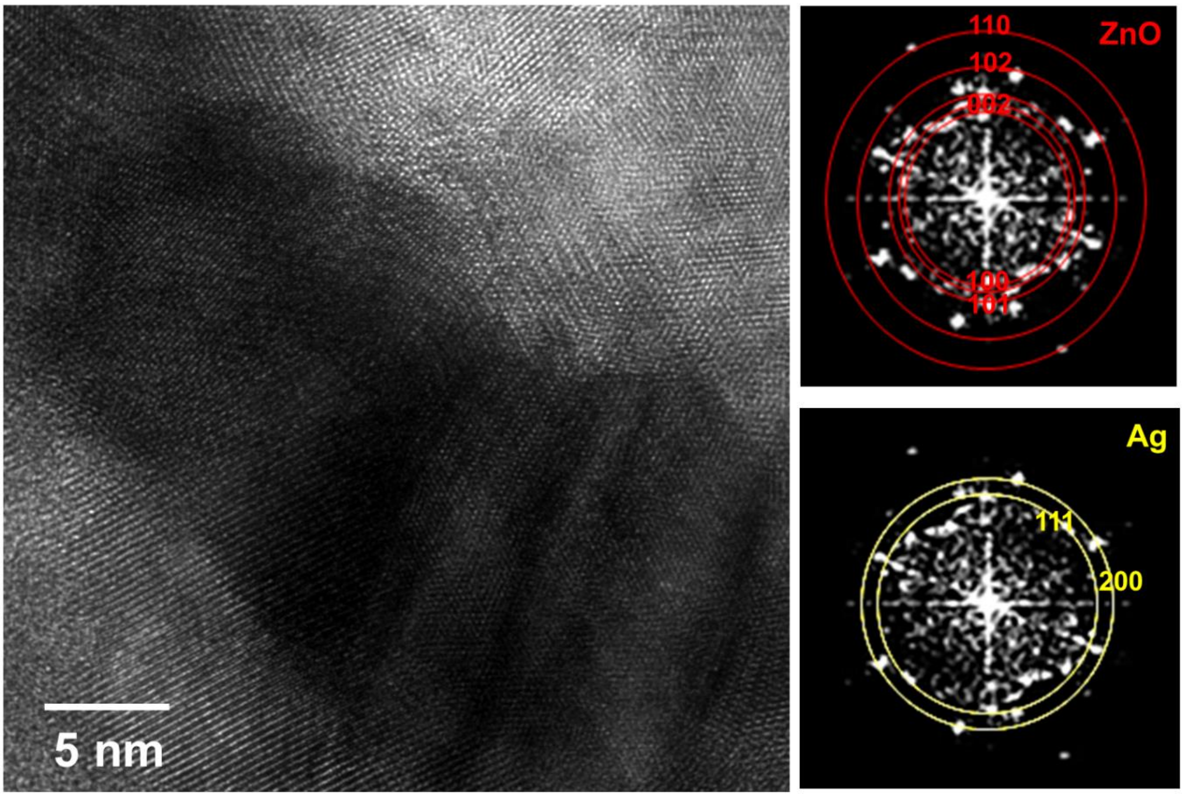

Figure S14. The HRTEM image and its FFT patterns of Ag conducting filaments in the LRS. The HRTEM image of the conducting filament corresponds to Fig. 3. The lower images are the FFT patterns of the red frame. The red rings represent the diffraction rings of ZnO, and the yellow rings indicate the diffraction rings of Ag. It can obviously be seen that several points are located in the Ag diffraction rings, demonstrating that the conducting filament is composed of Ag.
